# A semi-automatic motion-constrained Graph Cut algorithm for Pedestrian Detection in thermal surveillance videos

**DOI:** 10.7717/peerj-cs.1064

**Published:** 2022-09-12

**Authors:** Oluwakorede Monica Oluyide, Jules-Raymond Tapamo, Tom Mmbasu Walingo

**Affiliations:** Discipline of Electrical, Electronic, and Computer Engineering, University of KwaZulu-Natal, Durban, KwaZulu-Natal, South Africa

**Keywords:** Infrared video, Pedestrian detection, Video surveillance

## Abstract

This article presents a semi-automatic algorithm that can detect pedestrians from the background in thermal infrared images. The proposed method is based on the powerful Graph Cut optimisation algorithm which produces exact solutions for binary labelling problems. An additional term is incorporated into the energy formulation to bias the detection framework towards pedestrians. Therefore, the proposed method obtains reliable and robust results through user-selected seeds and the inclusion of motion constraints. An additional advantage is that it enables the algorithm to generalise well across different databases. The effectiveness of our method is demonstrated on four public databases and compared with several methods proposed in the literature and the state-of-the-art. The method obtained an average precision of 98.92% and an average recall of 99.25% across the four databases considered and outperformed methods which made use of the same databases.

## Introduction

Video surveillance technology is rapidly proliferating across public and private spaces. Traditionally, surveillance systems could only be found on buildings owned by the Government and large organisations. Currently, they can be found in a variety of settings such as shops, stadia, airports, schools and private residences. Two main factors are responsible for the ubiquity of video surveillance systems (VSS). The first is increased ease of acquisition and installation of VSS. This is due to the advancements in technology from analogue to digital systems and the significant drop in the cost of acquisition. The second factor is the increasing need for security globally. There is a high demand for persistent surveillance systems which can monitor round the clock all year round. As most VSS use visible-light cameras, the presence or absence of light hinders their ability to monitor persistently. Thermal cameras are viable substitutes because they function in poor lighting and at night. These cameras contain sensors which measure and create images from the thermal infrared energy emitted from objects in the scene (Negied, Hemayed & Fayek, 2015).

The amount of infrared detected determines how bright or how dark an object will appear in the final image. Emissivity is the ratio of infrared energy radiated from an object to that radiated from a perfect emitter under the same conditions. Given that 1 is the emissivity of a perfect emitter, also called a blackbody, pedestrians have a value of 0.98 (Fluke, 2020). Thermal imaging finds extensive application in pedestrian detection and tracking because pedestrians have high emissivity which creates a good enough contrast between them and the background. The challenge to detecting pedestrians in thermal images arises from the fact that, while pedestrians can emit infrared energy almost perfectly, only a fraction of the emissions are detected by the thermal camera. The amount of infrared energy reaching the thermal camera sensors depends on the prevailing weather conditions, the reflectivity of other objects in the scene and even the thermal camera itself. Thus, thermal images have lower resolution and lack the number of details present in visible-light images and the applications of thermal imaging are not as varied as those of visible imaging.

The motivation of this article is to propose a new method to detect pedestrians in thermal imaging acquired under different conditions. State-of-the-art algorithms for visible images usually do not perform with similar accuracy on thermal images and generally do not perform well across different datasets. This is because Image Analysis is slightly different when performed on visible and thermal images. Some of the characteristics of thermal images introduce additional challenges and/or nullify some steps in algorithms used for visible light images. For instance, there are immediate changes in appearance as illumination changes in visible images while appearance changes much slowly because detected radiation increases or decreases gradually in thermal images. Also, objects in thermal images do not cast shadows. Therefore, applying algorithms such as background subtraction to thermal images will not urgently need steps for scene update and shadow removal as will be the case for visible images. Furthermore, objects in visible images are commonly differentiated by their colour and displayed in the RGB (Red-Green-Blue) colour space while thermal information is commonly mapped to grayscale. It is important to remember that while RGB can be converted to grayscale, they still do not present the same information as thermal infrared images even if both images capture the same scene.

Furthermore, many of the methods put forward for pedestrian detection in thermal images require several steps grouped broadly into two: candidate generation and validation. Candidate generation involves extracting likely regions containing pedestrians. Candidate validation involves examining the extracted regions and discriminating between pedestrian and non-pedestrian. Errors tend to accumulate from each of these steps. Thus, different from other methods put forward, the proposed method is a single-model algorithm for pedestrian detection that eliminates the need for separate modules of candidate generation and validation. It integrates the appearance properties of the image with motion patterns such that all the fine-tuning and adjustment happens during energy formulation.

The contribution of this article is a novel Graph Cut energy function, referred to as motion-constrained energy (MCE), which repurposes binary segmentation for pedestrian detection in infrared images. Inspired by the semi-automatic framework of Boykov & Jolly (2001) that integrates the image region and boundary information into a single energy function, the proposed energy function incorporates an additional term to penalise pixels based on motion characteristics to accurately detect pedestrians in thermal images. The formulation in Boykov & Jolly (2001) presents an energy function *E* incorporating a region *D*(*h*) and boundary term *S*(*h*) shown as follows

(1) $$E(h) = \lambda \cdot \;D(h)\; + \;J(h)$$

where

$\matrix{ {D(h)} \hfill & { = \;\sum\limits_{y \in {\rm {\mathcal{y}}}} {{D_y}} ({h_y})} \hfill \cr {J(h)} \hfill & { = \;\sum\limits_{\{ y,z\} \in {\rm {\cal N}}} {{J_{y,z}}} ({h_y},{h_z}) \cdot {\epsilon }({h_y},{h_z})} \hfill \cr }$

and

${\epsilon }(a,b) = \left\{ {\matrix{ {1,} \hfill & {{\rm if}\;a \ne b} \hfill \cr {0,} \hfill & {{\rm otherwise}} \hfill \cr } } \right.$

where ${\rm {\cal N}}$ are unordered pairs of neighbouring pixels from a standard neighbourhood system *e.g*., 4-, 8- or 26- neighbourhood system and $\lambda$ is used to balance the contribution of the region and boundary term to the final segmentation result. *D*(*h*) measures how well pixels fit into the object or background models. *J*(*h*) is also called the smoothness term and it measures the similarity of intensity values between neighbouring pixels.

There are two areas where this formulation falls short in thermal images. Firstly, the low resolution and noisy nature of IR images mean that more importance will be given to the region term in many instances using this formulation. This means that a robust model for each class will have to be determined. As mentioned earlier, most models and approximated distributions in the literature do not generalise well across datasets, therefore, it is important to add another element to reduce over-dependence on the region term. Secondly, this formulation produces solutions where regions with similar intensity values as the pedestrians are included in the solution irrespective of their location.

The proposed energy function (MCE) incorporates motion constraints and is defined in Eq. (2) as

(2) $$E(h) = \;D(h)\; + \;J(h)\; + \;M(h)$$

where

$\matrix{ {D(h)} \hfill & { = \;\sum\limits_{y \in {\bf Y}} {{D_y}} ({h_y})\; \cdot \delta ({h_y})} \hfill \cr {J(h)} \hfill & { = \;\sum\limits_{\{ y,z\} \in {\bf N}} {{J_{y,z}}} ({h_y},{h_z}) \cdot {\epsilon }({h_y},{h_z})} \hfill \cr {M(h)} \hfill & { = \;\sum\limits_{y \in {\bf Y}} {{M_y}} ({h_y}) \cdot \beta ({h_y})} \hfill \cr }$

and

$\delta ({h_y}) = \left\{ {\matrix{ {1,} \hfill & {{\rm if}\;y \in {\rm {\mathcal{T}}} \wedge y\ \notin\ {\rm {\mathcal{D}}}comb} \hfill \cr {0,} \hfill & {{\rm otherwise}} \hfill \cr } } \right.$

${\epsilon }({h_y},{h_z}) = \left\{ {\matrix{ {1,} \hfill & {{\rm if}\;y \in {\rm {\mathcal{T}}} \wedge z \in {\rm {\mathcal{T}}} \wedge {h_y} \ne {h_z}} \hfill \cr {0,} \hfill & {{\rm otherwise}} \hfill \cr } } \right.$

$\beta ({h_y}) = \left\{ {\matrix{ {1,} \hfill & {{\rm if}\;y \in {\rm {\mathcal{D}}}comb} \hfill \cr {0,} \hfill & {{\rm otherwise}} \hfill \cr } } \right.$

where *M*(*h*) is the motion term, ${\rm {\mathcal{T}}}$ is the set of pixels containing one or more motion pixels and ${\rm {\mathcal{D}}}comb$ is the set of pixels with the highest energies from four directional difference images.

**Figure 1:**
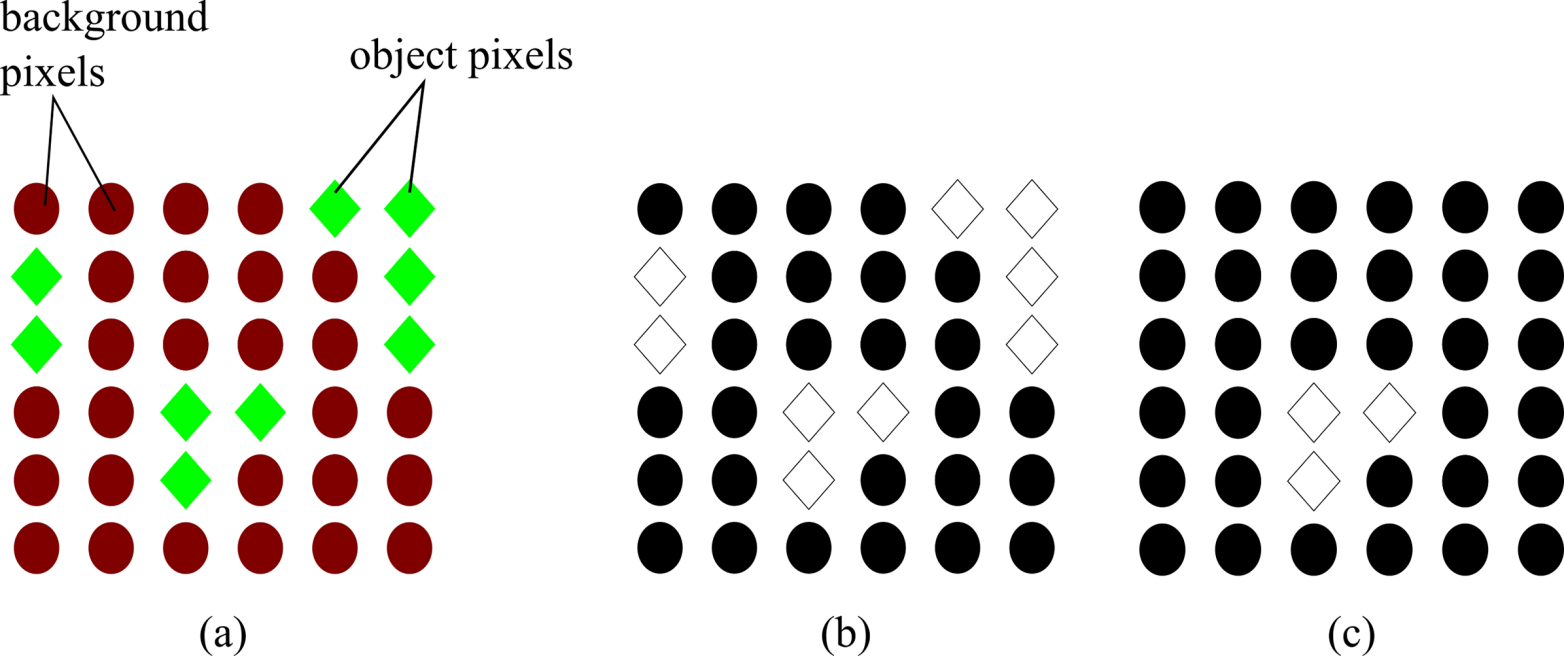
Difference between topologically unconstrained and constrained solution using Graph Cut (A) Original image showing the pixels belonging to the object of interest (green diamonds) and the background (red circles) (B) topological unconstrained solution: all the pixels with similar properties to the object of interest are included in the result (C) topological constrained solution: only the pixels of the object of interest are included in the result.

The impact of the proposed energy is expressed in Fig. 1. The result of using the energy of Boykov & Jolly (2001) produces topologically unconstrained solutions shown in Fig. 1B. This means that all pixels with the same properties as the object of interest will be included in the final result. However, MCE constrains the solution to only the object of interest as shown in Fig. 1C.

The rest of the article is organised as follows. Section 2 presents the related works. Section 3 presents the proposed framework. Section 4 provides the experimental results. Section 5 presents the conclusion and future work.

## Related works

The task of detecting pedestrians is necessary for understanding and recognising human activity and behaviour in video surveillance footage. In thermal infrared images, this task is carried out in two major steps. The first step is to detect all regions likely to contain pedestrians. This is called Candidate Generation. The second step is to discriminate from among the extracted regions those belonging to the pedestrians. This is called Candidate Validation.

Many methods put forward for candidate generation in thermal infrared images depend on the contrast between the pedestrian and background. Thresholding methods have, therefore, found extensive use in this domain and are into two categories: parametric and non-parametric. Parametric thresholding algorithms obtain a threshold by parameter estimation while non-parametric thresholding algorithms optimise an objective function. Examples of Parametric methods can be found in the works of Soundrapandiyan & Mouli (2015), Wu et al. (2017), Manda et al. (2020) and Manda & Kim (2020). Soundrapandiyan & Mouli (2015) proposed a method comprising background subtraction, high-boost filtering to highlight the pedestrian pixels and suppress the background before applying local adaptive thresholding. Wu et al. (2017) produced formulas to compute two threshold limits from the image histogram. One limit eliminates cold regions while the other eliminates extremely bright regions. To obtain the binary image, the pixels with intensity values between the low and high threshold limit are set to the maximum pixel intensity of 255 while all other values are set to 0. Manda et al. (2020) made use of the raised cosine distribution function to determine a threshold value which separates the pedestrian from the background. Manda & Kim (2020) assumed the distribution of the image follows that of the transient response of the first-order linear circuit to determine a threshold value for pedestrian detection. Non-parametric approaches can be found in the works of Li et al. (2011) and Wu, Hou & Chen (2016). Li et al. (2011) found that previous non-parametric methods proposed which perform well on visible images do not perform satisfactorily on thermal images because the object and background distributions are similar and proposed a new criterion for thresholding infrared images where the distribution for both classes have similar standard deviations. Wu, Hou & Chen (2016) proposed a new threshold criterion for infrared images by assuming normal distributions for both the object and background histograms and comparing the hyper-entropies of both distributions. Thresholding methods produce excellent results when the approximated distribution of the image fits the dataset under consideration. However, this means that they can easily become too dataset–dependent. Also, in situations where the contrast is not pronounced, the pedestrians are not of uniform appearance, or polarity reversal occurs due to change of weather and the presence of artefacts such as halos, detection based on appearance alone suffers setbacks.

To reduce dependence on the contrast for pedestrian detection, candidate generation has been carried out by detecting moving regions. Background Subtraction and Optical flow-based methods are commonly used for detecting moving regions, but Background Subtraction is less computationally expensive (Choudhury et al., 2018). Generally, Background Subtraction is carried out by creating a model of the image background and comparing that model with each video frame. A similarity function is employed to determine which pixels are likely to belong to the object of interest. Background Subtraction by Frame differencing detects moving regions and is commonly used in tracking algorithms (Gawande, Hajari & Golhar, 2020). The presence of motion can be obtained from the absolute difference between consecutive image pairs. Jeon et al. (2015) created a background model using pixel difference image and combined edge information with the result of background subtraction to detect the pedestrians. Jeyabharathi & Dejey (2018) made use of frame differencing to extract likely pedestrian regions and reflectional symmetrical patterns to provide geometrical information for accurate background modelling. Motion is one feature that can cut across a wide range of infrared images.

Candidate validation have be performed using unsupervised and supervised approaches. Unsupervised methods make use of known or calculated physical properties of the pedestrians to discriminate between pedestrian and non-pedestrian. Younsi, Diaf & Siarry (2020) proposed a global similarity function that uses the sum of sub-similarity functions to discriminate between human moving objects and non-human moving objects. The drawback of unsupervised methods is that they also tend to be data–dependent. Supervised methods depend on feature extraction and training. Although recent efforts are moving towards the use of Convolutional Neural Networks (CNN) where feature representation is an inherent part of the training framework, feature representation is still a challenge because thermal images have low resolution and fewer details compared with visible images. Recent efforts such as those of Dai et al. (2019), Park et al. (2019), Chen & Shin (2020), Gao, Zhang & Li (2020), Huda et al. (2020), Krišto, Ivasic-Kos & Pobar (2020), Tumas, Nowosielski & Serackis (2020) and Haider, Shaukat & Mir (2021) rely on features for a (pedestrian/non-pedestrian) classifier. Park et al. (2019) proposed a CNN-based classifier with three input channels for fine-grained pedestrian detection. The input channels take in the original image, a Difference image from the previous frame and a background subtraction mask. In their results, they noted that training and testing needed to be carried out on similar datasets for best performance. Chen & Shin (2020) developed an attention-guided autoencoder network that includes a skip-connection block which combines features from the encoder-decoder modules to increase contextual information for robust and distinguishable features in infrared images with low SNR and resolution. YOLOv3 was used by Krišto, Ivasic-Kos & Pobar (2020) and Tumas, Nowosielski & Serackis (2020) for pedestrian detection under different weather conditions. Gao, Zhang & Li (2020) redesigned the visual geometry group (VGG-19) CNN to extract more features from infrared images for better detection results. The rationale for using these methods is that they perform well on visible images and achieve state-of-the-art results. However, their performance is lower on infrared images for two reasons. First, the models developed by Huda et al. (2020) for testing infrared images were trained on visible images. Second, different thermal cameras output different levels of detail. Therefore, even for models trained on infrared images such as done by Krišto, Ivasic-Kos & Pobar (2020) and Park et al. (2019), the performance of the trained model depends on how similar the test dataset is to the training dataset.

To the best of our knowledge, semi-automatic methods requiring human inputs have not yet found extensive application in the thermal domain. This work is inspired by the methods put forward by Boykov & Jolly (2001) and Viola, Jones & Snow (2003). Graph Cut is a powerful optimization method that guarantees an exact solution for binary labelling problems. Graph Cut’s effectiveness is shown in the framework of Boykov & Jolly (2001) which seamlessly combines edge and appearance information into its energy formulation to produce topologically unrestrained solutions Boykov & Funka-Lea (2006). This means that all pixels with the same properties are given the same label regardless of their location. Viola, Jones & Snow (2003) proposed a method which eliminates the need for separate modules for pedestrian detection and put forward a detector that integrates appearance and motion patterns such that all the fine-tuning and adjustment happens during training. Both methods are similar in that they seamlessly combine different attributes to accomplish one goal that would otherwise have required several steps. Also, both methods were tested and achieved state-of-art on visible images. However, the framework of Boykov & Jolly (2001) is semi-automatic while that of Viola, Jones & Snow (2003) is supervised.

## Proposed method

**Figure 2:**
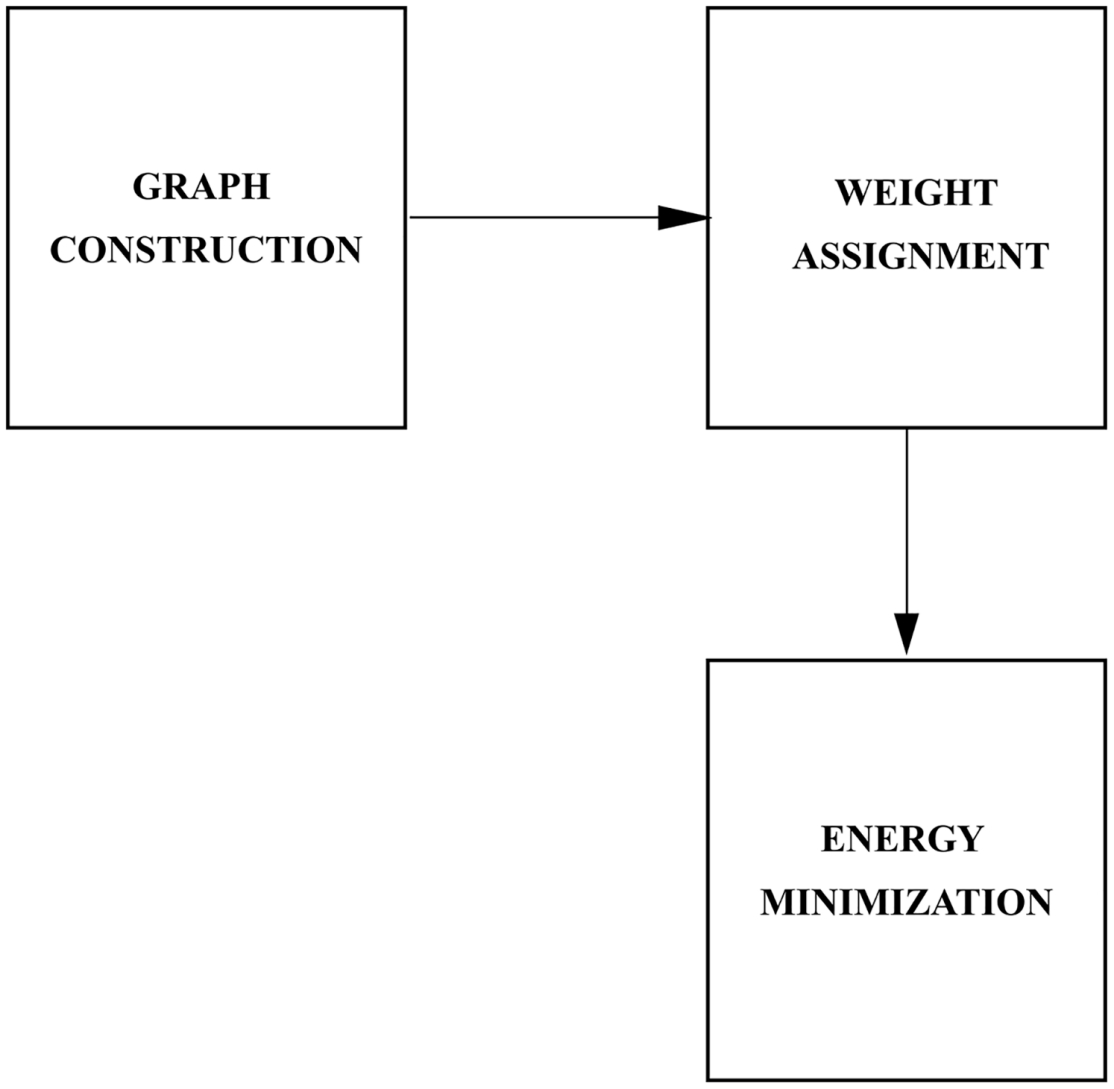
Overview of the proposed method.

This work considers a Graph-Cut based method for pedestrian detection which combines intensity (region and boundary) information with motion characteristics. The task of pedestrian detection is formulated as a binary labelling problem where the goal is to partition the image into two classes. Formally, the labelling problem is a function that maps observed data to labels. For our purposes, the observed data is the image and the labels are the classes. Let labels ${\rm {\mathcal{Z}}}$ assigned to a pixel be given as ${\rm {\mathcal{Z}}} = ($‘ped’, ‘bkg’) where ‘ped’ refers to the ROI and ‘bkg’ refers to the rest of the scene. The labelling of ${\rm {\mathcal{X}}}$ over ${\rm {\mathcal{Z}}}$ is a function $h:{\rm {\mathcal{X}}} \to {\rm {\mathcal{Z}}}$. ${h_x}$ specifies the label assignments to *x* in ${\rm {\mathcal{X}}}$ and is taken from ${\rm {\mathcal{Z}}}$. To solve the binary labelling problem, Graph Cut performs efficient searches for the optimal labels among the possible set of labels. A graph is constructed over the image and a cut on the graph corresponds to the binary partitioning of the image. An energy function is used to represent the information in the image and the global minimum of the energy corresponds to the optimal partitioning. The overview of the proposed method is presented in Fig. 2.

### Graph construction

**Figure 3:**
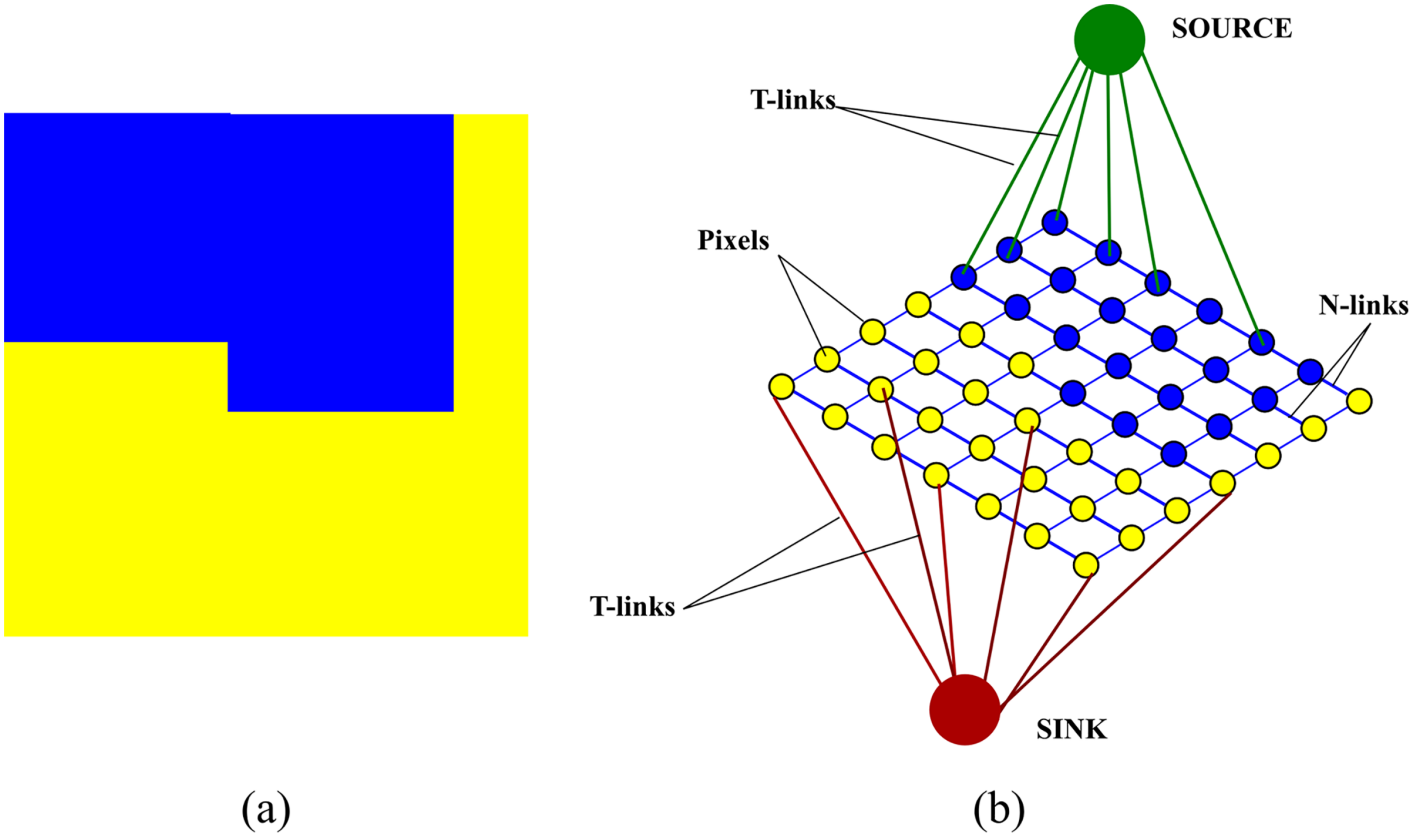
An illustration of a graph constructed over an image (A) original image (B) graph construction.

The first step is to construct a graph ${\rm {\cal G}}$ over an image. ${\rm {\cal G}} = \langle {\rm {\cal V}},{\rm {\cal E}}\rangle$ where ${\rm {\cal V}}$ are the nodes of the graph and ${\rm {\cal E}}$ are the edges. ${\rm {\cal V}}$ correspond to the pixels of the image and include two additional nodes, source *s* and sink *t*, called terminals. The edges which connect the pixels to each other are referred to as N-links while the edges which the pixels to the two terminals are referred to as T-links. A neighbourhood system **N** determines the placement of edges between the nodes. A non-negative weight, discussed in “Weight assignment”, is assigned to each edge. An illustration of a graph constructed over an image is shown in Fig. 3

### Weight assignment

The non-negative weights for each edge edge $e \in {\rm {\cal E}}$ of the graph ${\rm {\cal G}}$ are calculated from the region, boundary and motion terms of Eq. (2).

The region term, ${D_y}({h_y})$ reflects the extent to which each pixel fits into the image intensity model of “object” and “background”. These weights, ${D_y}($“object”) and ${D_y}($“background”), are computed as negative log-likelihoods as follows.

(3) $$\matrix{ {{D_y}({''} {\rm object{''} })} \hfill & { = - \ln {\rm Pr}({Y_y}|{''} {\bf object}{''} )} \hfill \cr {{D_y}({''} {\rm background{''} })} \hfill & { = - \ln {\rm Pr}({Y_y}|{''} {\bf background}{''} )} \hfill \cr }$$

The intensity model for ${D_y}({h_y})$ is built using pixels, called seeds, which definitely belong to the “object” and “background”. These seeds are chosen interactively by the user.

The boundary term ${J_{y,z}}({h_y},{h_z})$ assigns penalties to discontinuities between neighbouring pixels *y* and *z*. Therefore, the edge weights between pixels with dissimilar pixel intensity values will be higher and *vice versa*. These weights are calculated as follows

(4) $${S_{y,z}} = \exp \left( {\displaystyle{{{{(y - z)}^2}} \over {2{\sigma ^2}}}} \right)$$

In the above equation, $\sigma$ has been calculated as the variance of the video frame under consideration.

The motion term ${M_y}({h_y})$ computes the cost of labelling a pixel as “object” or “background” as determined by the motion constraint ${\rm {\mathcal{D}}}comb$. ${\rm {\mathcal{D}}}comb$ provides an estimate of the location of each pedestrian in the image obtained by thresholding and combining four images obtained by frame differencing. The direction of motion can be obtained from the absolute differences ${\rm {\mathcal{D}}}{L_f}$, ${\rm {\mathcal{D}}}{R_f}$, ${\rm {\mathcal{D}}}{U_f}$ and ${\rm {\mathcal{D}}}{D_f}$ between consecutive image pairs ${I_f}$ and shifted versions of ${I_{f + 1}}$ to the left, to the right, up and down respectively. The difference image computations are given as follows

(5) $$\matrix{ {{{\rm {\mathcal{D}}}_f}} \hfill & { = |{I_f} - {I_{f + 1}}|} \hfill \cr {{\rm {\mathcal{D}}}{L_f}} \hfill & { = |{I_f} - {I_{f + 1}} \leftarrow |} \hfill \cr {{\rm {\mathcal{D}}}{R_f}} \hfill & { = |{I_f} - {I_{f + 1}} \to |} \hfill \cr {{\rm {\mathcal{D}}}{U_f}} \hfill & { = |{I_f} - {I_{f + 1}} \uparrow |} \hfill \cr {{\rm {\mathcal{D}}}{D_f}} \hfill & { = |{I_f} - {I_{f + 1}} \downarrow |} \hfill \cr }$$

**Figure 4:**
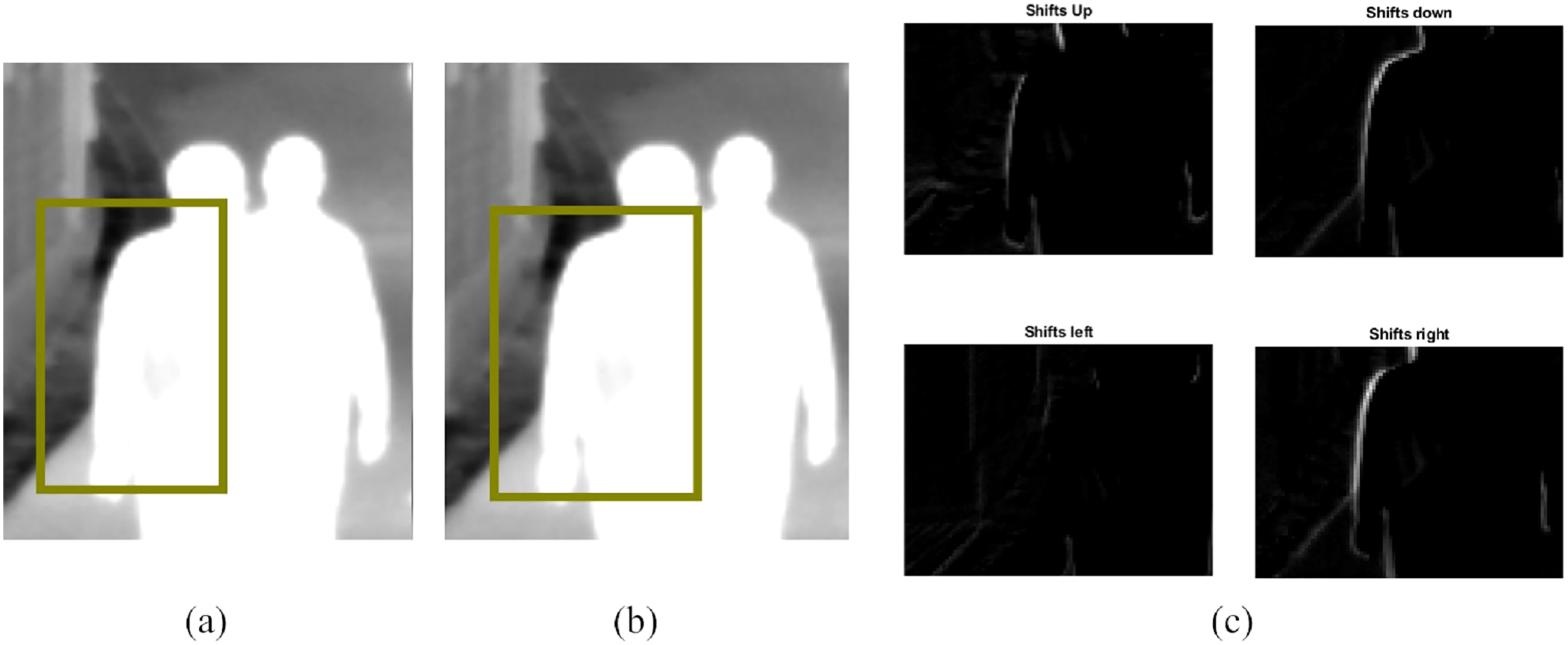
(A) and (B) are two consecutive frames with an area of interest selected and (C) shows the directional difference images around that selected area. The image energy is higher when the image is shifted to the right than to the left, and then when it is shifted downwards than upwards. So, without previous knowledge, we can tell the pedestrian is moving to the right and slightly downwards. Image credit: LITIV dataset; Torabi, Massé & Bilodeau (2012).

Figure 4 shows how the shifted difference images provide information about the direction of motion. In our experiments, we found that the energy of the image was highest when the image was shifted in the direction of motion and the least when shifted in the opposite direction. Also, because the surveillance footage is taken from different angles and there are usually several pedestrians going in different directions, we found that the energy for each subject is higher in at least two directions, that is, either in the *↑* or *↓* direction and either in $\leftarrow$ or $\to$ direction.

${\rm {\mathcal{D}}}comb$ is, therefore, created by combining the pixels with the highest energies from each directional difference image and is defined as

(6) $${\rm {\mathcal{D}}}comb = \{ ({\rm {\mathcal{D}}}{L_f} > {\rm Th}) \cup ({\rm {\mathcal{D}}}{R_f} > {\rm Th}) \cup ({\rm {\mathcal{D}}}{U_f} > {\rm Th}) \cup ({\rm {\mathcal{D}}}{D_f} > {\rm Th})\}$$

where Th is used to extract the highest energies from each directional difference image.

${M_y}({h_y})$ is, thus, defined as follows

(7) $${M_y}(''{\rm object{''}}) = \left\{ {\matrix{ {\eta ,\;\;\;{\rm if}\;\;y \in {\rm {\mathcal{D}}}comb} \hfill \cr {0,\;\;{\rm otherwise}} \hfill \cr } } \right.$$

(8) $${M_y}(''{\rm background{''}}) = \left\{ {\matrix{ {\eta ,\;\;\;{\rm if}\;\;y\ \notin\ {\rm {\mathcal{D}}}comb} \hfill \cr {0,\;\;{\rm otherwise}} \hfill \cr } } \right.$$

where $\eta$ is an arbitrarily large number to ensure that the object or background label is assigned to a pixel if the stated condition for each class of assignment is satisfied.

Table 1 provides the weights for the graph edges. As discussed in “Graph construction”, the elements of ${\rm {\cal V}}$ for graph ${\rm {\cal G}}$ are the image pixels. Each node, corresponding to pixel *y*, is connected to the source *s* and sink *t* terminals using edges {*y*, *s*} and {*y*, *t*} called T-links. Also, each node is connected to other nodes in its neighbourhood. A four-neighbourhood system, for example, would mean that a pixel was connected to its four neighbours above, below, to the left and the right of it. The edges which connect a node to its neighbours {*y*, *z*} are called N-links. A higher weight on the T-link connecting a node to either *s* or *t* implies a higher likelihood of a pixel to be labelled as “object” or “background” respectively. Likewise, a higher weight on the N-link between vertices implies a greater dissimilarity between pixels. It should be noted that ${D_y}({h_y})$ and ${M_y}({h_y})$ are unary terms acting on each pixel to compute the weight on the T-links of the graph while ${J_{y,z}}({h_y},{h_z})$ is a binary term acting on pixel pairs *y* and *z* in a specified neighbourhood **N** to compute the weight on the N-links.

**Table 1:** Edge weights of the graph constructed from the image.

Edge	Weight	For
{*y*, *z*}	${J_{y,z}}$	$\{ y,z\} \in {\bf N},y \in {\rm {\mathcal{T}}} \wedge z \in {\rm {\mathcal{T}}}$
{*y*, *s*}	${D_y}({\rm {''}pedestria{n}{''}})$	$y \in {\bf Y},y \in {\rm {\mathcal{T}}}$
	${M_y}({\rm {''}pedestria{n}{''}})$	$y \in {\bf Y},y \in {\rm {\mathcal{D}}}comb$
	0	$y \in {\bf Y},y\ \notin\ {\rm {\mathcal{D}}}comb \wedge y\ \notin\ {\rm {\mathcal{T}}}$
{*y*, *t*}	${D_y}({\rm {''}backgroun{d}{''}})$	$y \in {\bf Y},y\ \notin\ {\rm {\mathcal{T}}}$
	${M_y}({\rm {''}backgroun{d}{''}})$	$y \in {\bf Y},y\ \notin\ {\rm {\mathcal{D}}}comb$
	0	$y \in {\bf Y},y\ \notin\ {\rm {\mathcal{D}}}comb \wedge y\ \notin\ {\rm {\mathcal{T}}}$

To obtain ${\rm {\mathcal{T}}}$, the image is divided into non-overlapping equal-sized detection windows such that only windows which have one or more pixels from ${\rm {\mathcal{D}}}comb$ are considered by *D*(*h*) and *S*(*h*).

### Energy minimization

**Figure 5:**
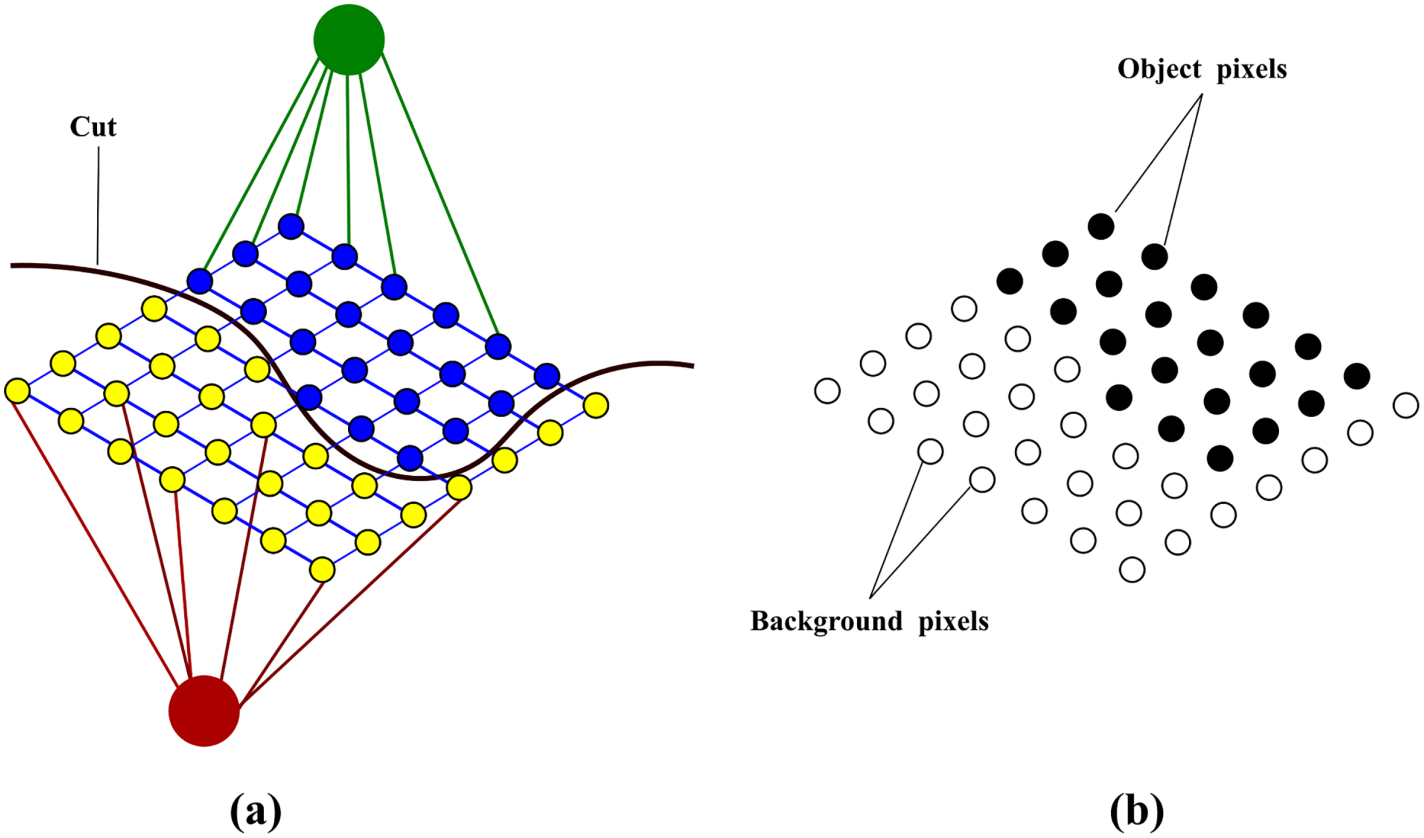
Binary labelling of an image after energy minimization (A) shows the minimum cut separating the vertices (B) shows the binary labelling as a result of the cut.

Following the graph construction and weight assignment, the energy is minimized using the Boykov-Kolmogorov minimum cut/maximum flow algorithm (Boykov & Kolmogorov, 2004). The aim of this algorithm is to find the cut ${\rm {\cal C}}$ that partitions a two-terminal graph into two disjoint sets *S* and *T* such that *s* is in *S* and *t* is in *T*. The optimization problem, to find the minimum among all possible cuts, is solved by finding the maximum flow moving from the source *s* to the sink *t*. The cost of the cut ${\rm {\cal C}} = \{ S,T\}$ is the sum of the weights on the edges (*y*, *z*) where *y* ∈ *S* and *z* ∈ *T*. The final labelling on the original image is produced by the minimum cut separating the two terminals shown in Fig. 5.

## Experimental results

### Dataset

The proposed method is tested on the following public databases as previously described in Oluyide, Tapamo & Walingo (2022):

1. The Linkoping Thermal InfraRed (LTIR) dataset put forward by Berg, Ahlberg & Felsberg (2015)
2. LITIV dataset put forward by Torabi, Massé & Bilodeau (2012)
3. OTCBVS benchmark – Terravic Motion IR database put forward by Miezianko (2005)
4. OTCBVS benchmark – Ohio State University (OSU) thermal pedestrian database put forward by Davis & Keck (2005)

### Performance metrics

The performance of the proposed method is measured using Recall and Precision given in Eqs. (9) and (10).

(9) $${\rm Recall} = \displaystyle{{TP} \over {TP + FN}}$$

(10) $${\rm Precision} = \displaystyle{{{\rm TP}} \over {{\rm TP + FP}}}$$

### Comparison of MCE with energy function of Boykov & Jolly (2001)

**Figure 6:**
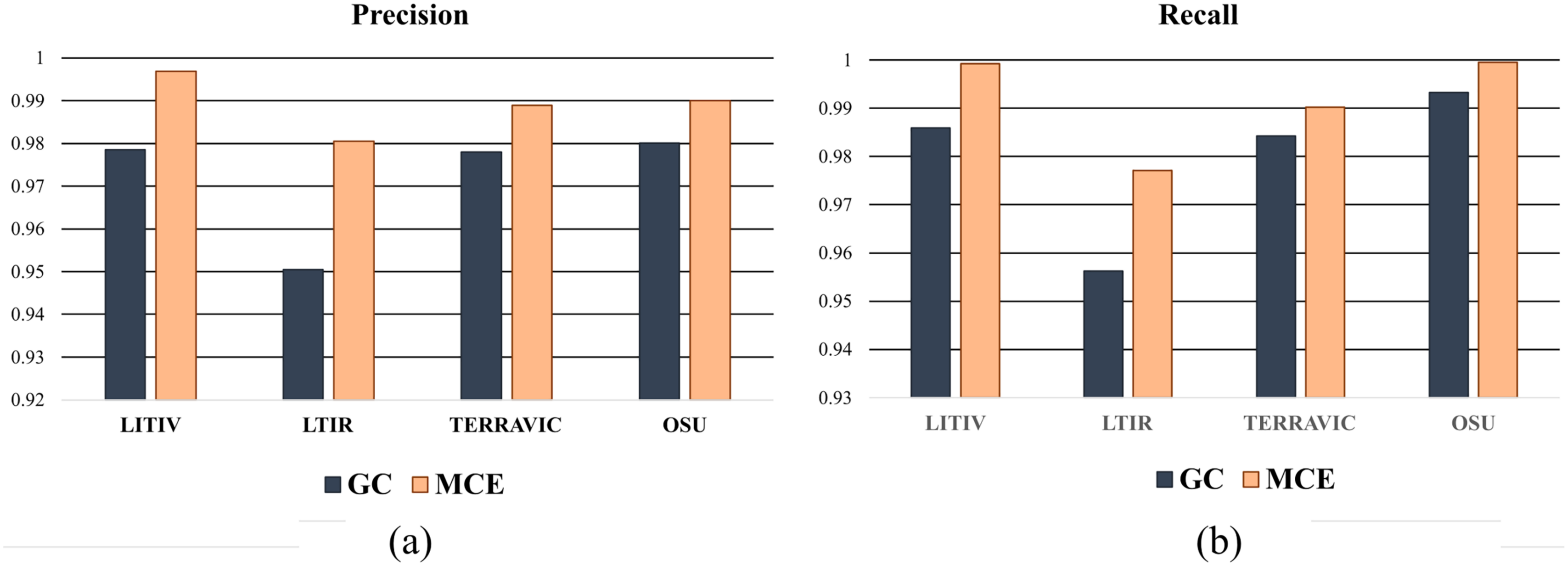
Chart showing the performance of GC and MCE using precision and recall.

**Figure 7:**
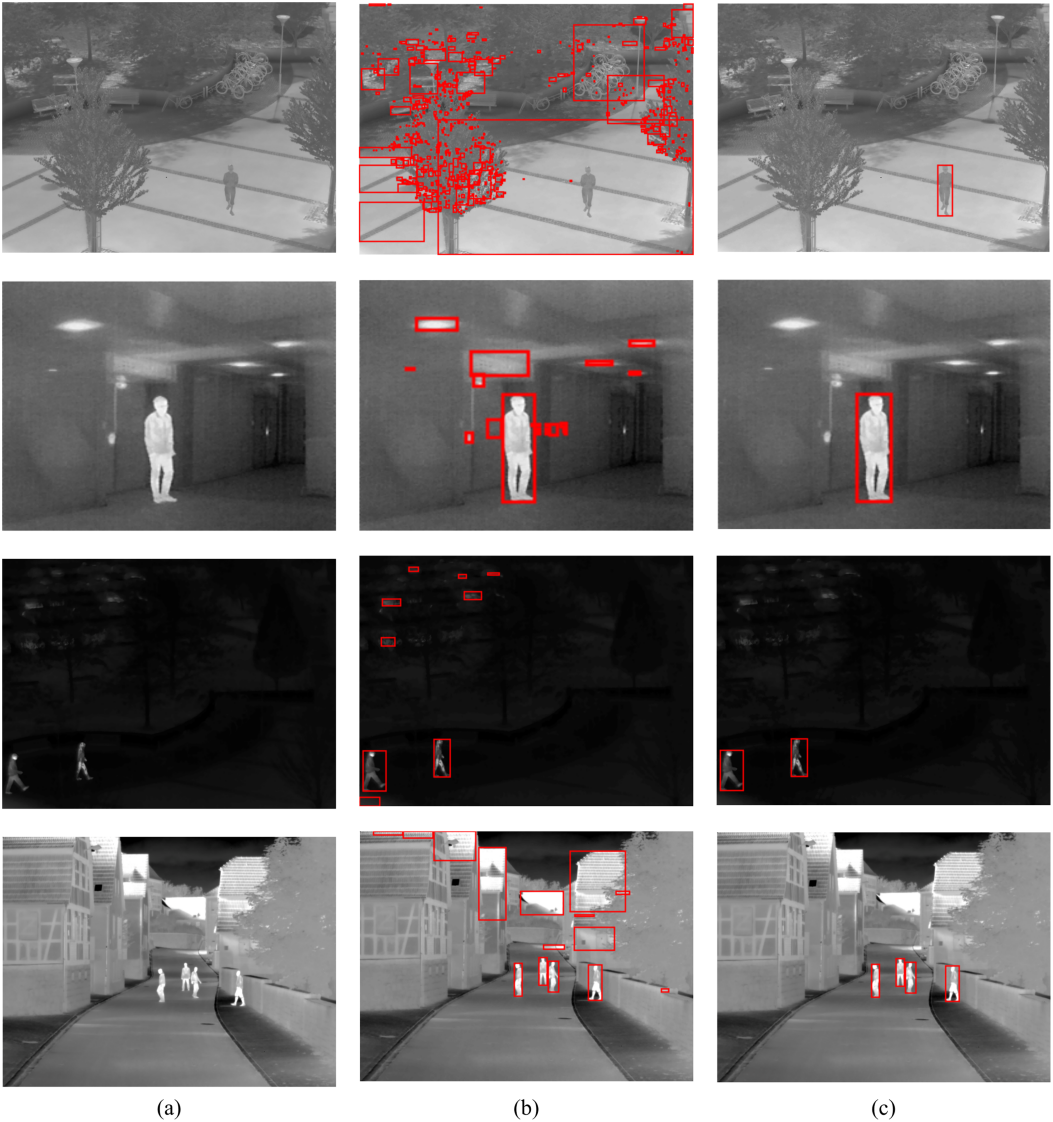
GC and MCE results on LTIR database (A) image (B) GC (C) MCE. Image credit: Linkoping Thermal InfraRed (LTIR) dataset; Berg, Ahlberg & Felsberg (2015).

**Figure 8:**
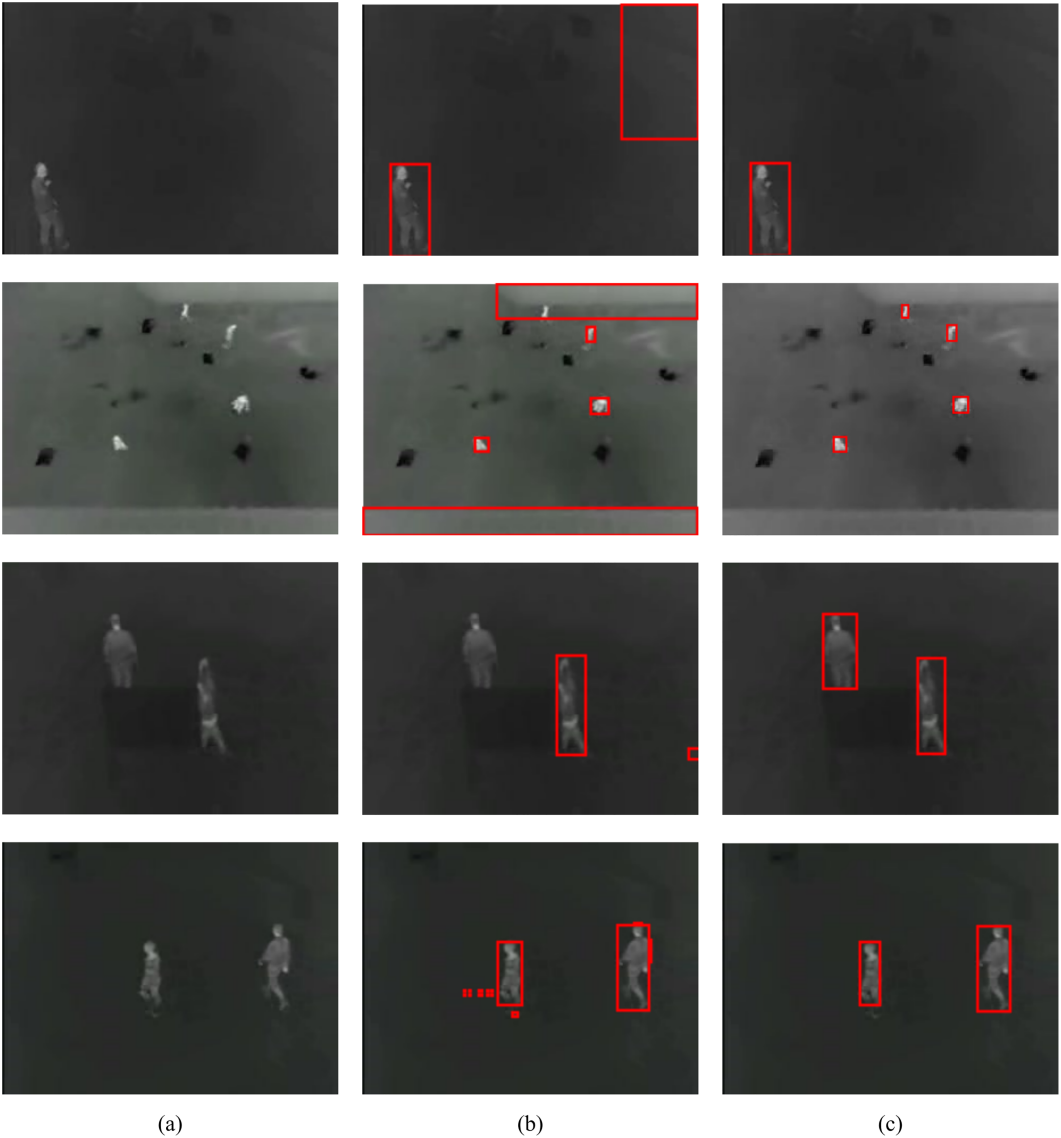
GC and MCE results on LITIV database (A) image (B) GC (C) MCE. Image credit: Linkoping Thermal InfraRed (LTIR) dataset; Berg, Ahlberg & Felsberg (2015).

**Figure 9:**
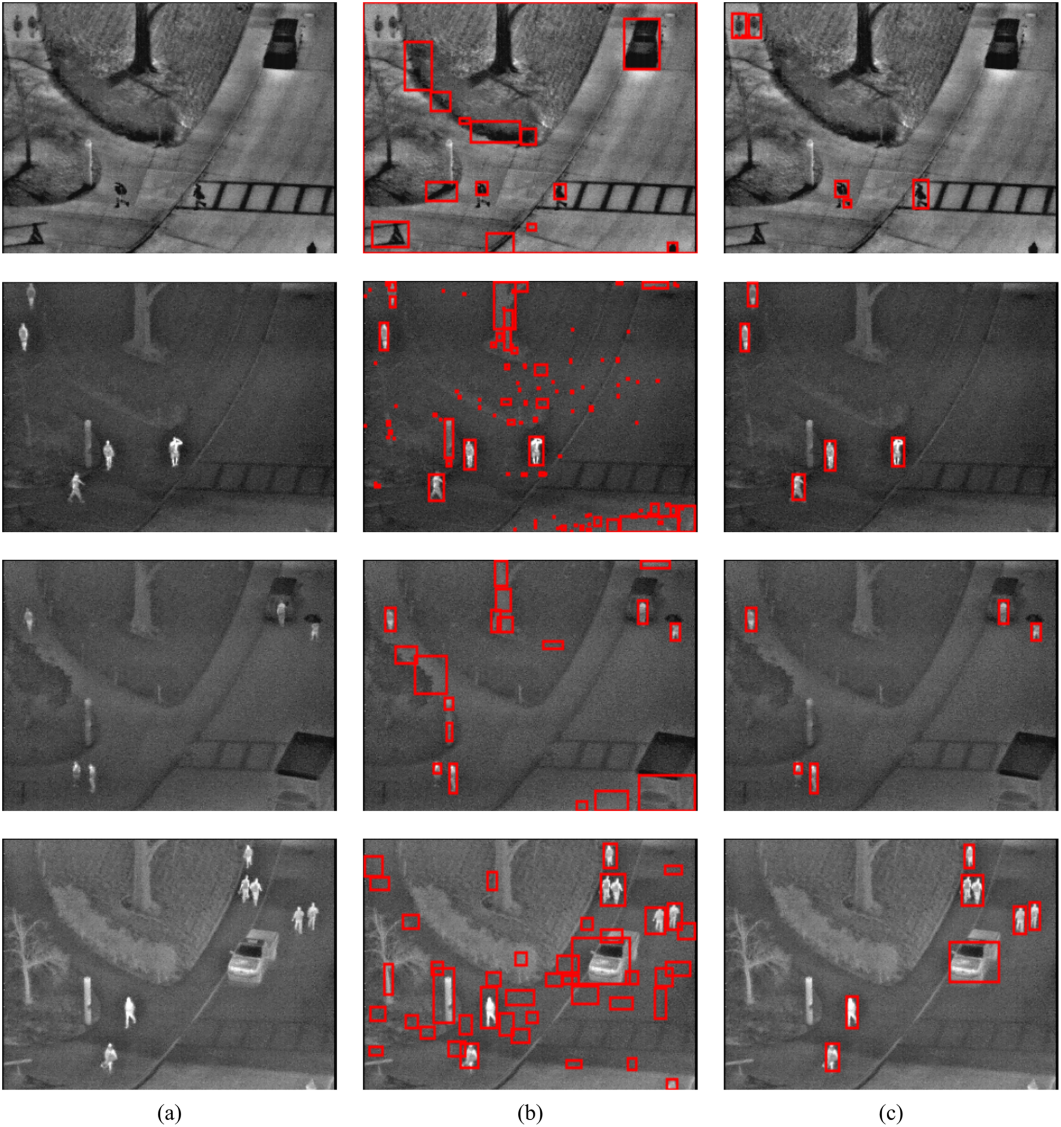
GC and MCE results on OSU database (A) image (B) GC (C) MCE. Image credit: Linkoping Thermal InfraRed (LTIR) dataset; Berg, Ahlberg & Felsberg (2015).

**Figure 10:**
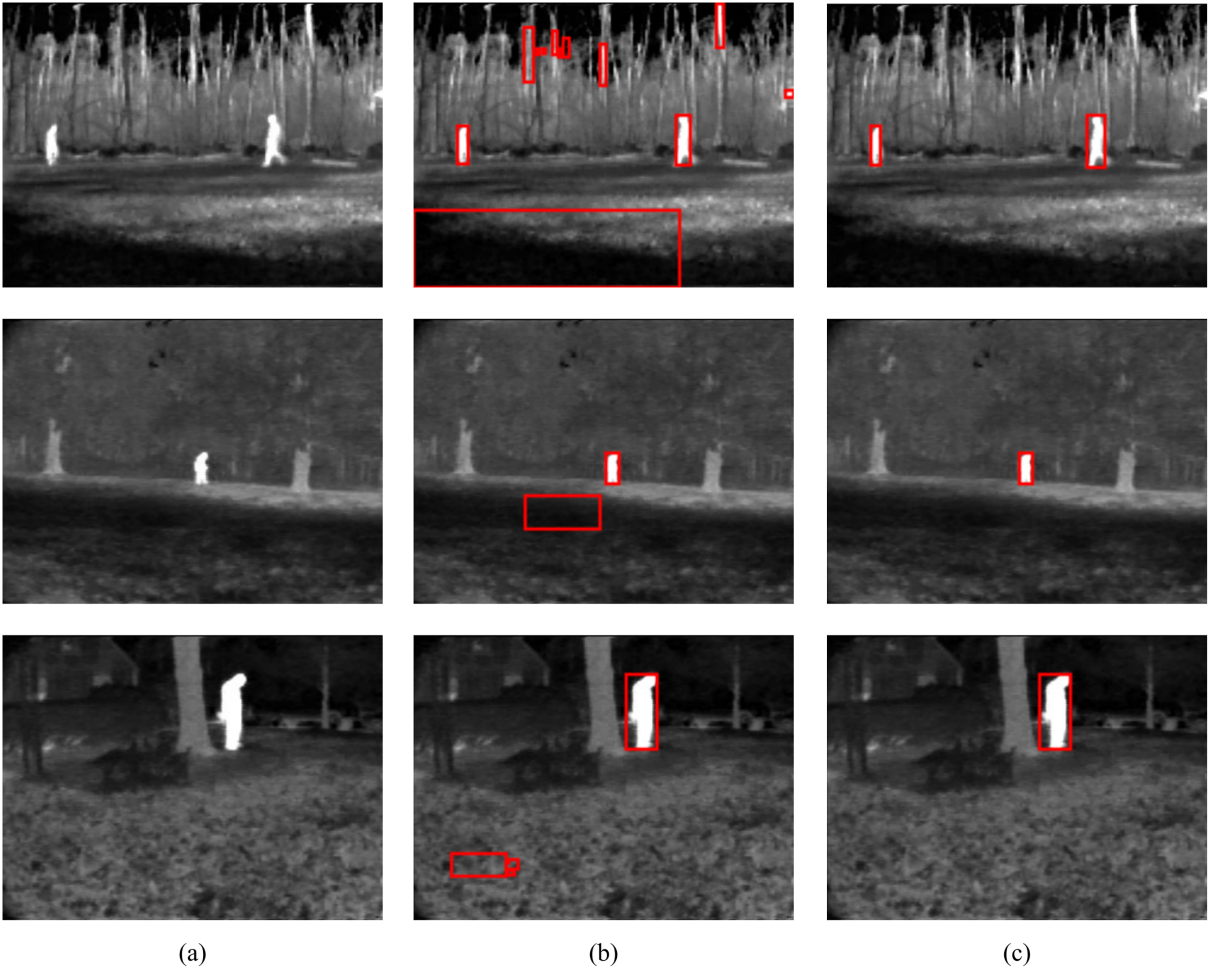
MCE results on TMIR database (A) image (B) GC (C) MCE. Image credit: Linkoping Thermal InfraRed (LTIR) dataset; Berg, Ahlberg & Felsberg (2015).

To show the improvements of the proposed method by the inclusion of an additional term in the energy function, the detections results of the proposed method (MCE) are compared with those produced by the energy of Boykov & Jolly (2001) (GC). The results of the comparison are presented in Table 2 and Fig. 6. The visual comparison of GC and MCE is shown in Figs. 7–10.

**Table 2:** Quantitative results for GC and MCE comparison.

	Method	LITIV	LTIR	Terravic	OSU
Recall	GC	0.9859	0.9562	0.9842	0.9933
	MCE	0.9992	0.9771	0.9902	0.9995
Precision	GC	0.9785	0.9504	0.9780	0.9801
	MCE	0.9969	0.9805	0.9889	0.9907

The performance of both GC and MCE is lowest on the LTIR database. This could be because LTIR has the most varied scenes of all the datasets. The images were either too bright or too dark and there were cases of slight camera motion and reversed polarity. Conversely, it shows the greatest improvement in performance when MCE is used.

LITIV database has the most uniform appearance but is the most varied in perspective; images were captured from different angles from the side view to the top view. Most of the images were very dark and the contrast was poor except in images taken from the top view. Significant improvement in performance is also observed when MCE is used.

The Terravic database had the best contrast, but the pedestrians were not always moving and, compared to the other databases, it took a long time for the pedestrians to move significantly. The impact of this slow or lack of movement is in deciding the interval between consecutive frames. Ideally, the next immediate frame should be used but this might depend on the footage.

The OSU database is the oldest and most extensively used because it was created specifically for evaluating pedestrian detection algorithms. The database contains details about the weather condition and comprehensive ground truth. The images were taken over different days and under different weather conditions but from the same scene. As mentioned in the introduction, temporal changes in appearance do not occur in thermal images unless there is a drastic change in weather conditions, and these changes occur much slowly as detected radiation increases or decreases gradually. Table 3 shows the weather conditions for each video sequence in the database, the total number of pedestrians in the database and the true positive (TP) and false positive (FP) detection results using the proposed algorithm. It can be concluded that the proposed method is quite robust to changes in weather.

**Table 3:** Table showing the weather conditions, true positive (TP) and false positive (FP) detection results for the OSU thermal database.

Video	Atmospheric phenomenon	Time of day	Temp (°C)	Total pedestrians	TP	FP
1	Light rain	Afternoon	13	91	90	0
2	Partly cloudy	Morning	5	100	98	2
3	Partly cloudy	Morning	21	101	98	3
4	Fair	Morning	9	109	109	0
5	Partly cloudy	Morning	25	101	99	1
6	Mostly cloudy	Morning	21	97	95	0
7	Light rain	Afternoon	36	94	92	1
8	Light rain	Afternoon	30	99	95	0
9	Haze	Afternoon	18	95	95	1
10	Haze	Afternoon	23	97	90	1
				984	961	9

### Comparison with other methods in the literature

The performance of the proposed method is presented in comparison with other methods in the literature (Table 4). Tables 5 and 6 compare the number of True Positive (TP) and False Positive (FP) detections obtained by the proposed method with other methods which use the OSU dataset including the creator of the Dataset Davis & Keck (2005). In Tables 5 and 6, the best result(s) for each sequence from each author is highlighted in bold. It is important to note that Sequence 3 has its polarity reversed, therefore, the pedestrians appear dark. Thus, Manda et al. (2020) do not provide results for sequence 3 because their method is for detecting bright regions in thermal images. While the proposed method does not always produce the best result for each sequence in Table 5, the average results outperform the methods put forward.

**Table 4:** Legend for Tables 5 and 6 column names.

Author	Letter
Davis & Keck (2005)	A
Wu et al. (2017)	B
Soundrapandiyan & Mouli (2018)	C
Zhao et al. (2019)	D
Manda et al. (2020)	E

**Table 5:** Comparing the proposed method with other methods using the number of true positive (TP) detections on the OSU dataset.

Video	#Pedestrians	A	B	C	D	E	MCE
1	(91)	88	90	87	77	78	90
2	(100)	94	95	96	99	98	98
3	(101)	101	101	83	64	-	98
4	(109)	107	108	109	107	109	109
5	(101)	90	95	100	97	101	97
6	(97)	93	94	94	92	97	93
7	(94)	92	93	86	78	80	90
8	(99)	75	80	97	89	96	93
9	(95)	95	95	95	91	95	95
10	(97)	95	95	94	91	83	89
1–10	(984)	930	946	941	885	829	961

**Table 6:** Comparing the proposed method with other methods using the number of false positive (FP) detections on the OSU dataset.

Video	A	B	C	D	E	MCE
1	0	0	5	3	0	0
2	0	0	14	2	2	2
3	1	1	27	90	-	3
4	1	0	18	7	10	0
5	0	0	13	16	16	1
6	0	0	2	8	0	0
7	0	0	4	8	0	1
8	1	1	3	8	0	0
9	0	0	2	4	0	1
10	3	3	8	18	16	1
1–10	6	5	96	164	44	9

The proposed method is also compared with methods which apply the state-of-the-art algorithms for object detection in visible images to thermal images using Precision and Recall. Table 7 presents the results of this comparison. As mentioned in “Related works”, the low performance of the state-of-the-art is because the models were either trained on visible images or trained on datasets dissimilar to the test set. However, the proposed method performs well across the different datasets.

**Table 7:** Comparison of the proposed method with the state-of-the-art using precision and recall.

Author	Metrics	LITIV	LTIR	Terravic	OSU
Lahouli et al. (2018)	Precision	0.9679	–	–	0.9737
	Recall	0.7819	–	–	0.7375
Krišto, Ivasic-Kos & Pobar (2020)	Precision	–	0.6700	0.9600	0.8600
	Recall	–	0.7500	0.9500	0.8900
Huda et al. (2020)	Precision	–	–	–	0.7100
	Recall	–	–	–	0.6100
Haider, Shaukat & Mir (2021)	Precision	–	–	–	0.9920
	Recall	–	–	–	0.9775
Proposed method	Precision	0.9969	0.9805	0.9899	0.9907
	Recall	0.9992	0.9771	0.9902	0.9995

### Time complexity and execution time

The steps of the proposed method are given in Algorithm 1. The time complexity can be determined as follows. In step 1, the directional difference images are computed using Eq. (5) and each computation takes O(*n*) time. In step 2, the location estimate image is computed using Eq. (6) and it involves two stages: finding the highest energies in each difference image and creating a new image from the union of the highest energies. Each stage is computed in O(*n*) time. Computing the edge weights using Table 1 is the third step and it involves the use of two matrices; an adjacency matrix for the N-links and an *nx*2 matrix for the T-links. For the adjacency matrix, adding a node takes O( ${n^2}$) time, adding an edge takes O(1) time and finding neighbours takes O(*n*) time. The overall time for the adjacency matrix is O( ${n^2}$). In the *nx*2 T-links matrix, one column holds the weights for pixels connected to the Source terminal and the second column holds the weights for pixels connected to the Sink terminal. Computing the weights for each terminal takes O(*n*) time. Thus, the overall time for step 3 is O( ${n^2}$). In step four, the minimization algorithm has a worst-case time complexity of O( $m{n^2}|C|$) where *n* is the number of nodes, *m* is the number of edges and |*C*| is the cost of the minimum cut. This algorithm outperforms standard minimization algorithms on typical Computer Vision problems even though the complexity of the algorithm is theoretically worse. The reader is referred to the work of Boykov & Kolmogorov (2004) for more details. Therefore, the overall time complexity of the proposed method is O( $m{n^2}|C|$).

**Algorithm 1:** Motion-constrained Graph Cut.

1: Compute the directional difference images using Eq. (5)
2: Compute the location estimate map ${\mathcal{D}}{comb}$ using Eq. (6)
3: Compute edge weights according to Table 1
4: Minimise the energy using Boykov-Kolmogorov min-cut/max-flow algorithm

The proposed method was implemented using MATLAB R2018a $^{TM}$ on an Intel i7-4790 3.60 GHz CPU with 8 GB RAM. The average execution time for each video frame ranged from 6.8 to 11.3 s depending on how fast the user selects seeds.

### Limitations of the proposed method

The main limitation which potentially reduces the effectiveness of the proposed method is the presence of extreme camera motion. A bit of camera motion was encountered in the LTIR database which can account for its lower performance compared to the other four datasets. However, if it is extreme, then it can hamper the effectiveness of the difference images produced using Eq. (5) because stationary objects might be included in the results. Although there are methods to correct camera motion, the additional step implies increased computational cost.

## Conclusion

In this article, a motion-constrained Graph Cut framework for pedestrian detection in thermal infrared videos has been presented which integrates appearance information with motion characteristics in a single model. The proposed method has been compared with the framework of Boykov & Jolly (2001) to show the advantages of including an additional constraint and the performance of the detection framework. In addition, the method has been tested on four publicly available datasets and with different methods in the literature which make use of the same datasets to showcase the robustness of the framework. As the process of selecting seeds significantly increases the execution time, future work will involve optimising the algorithm to require as little human input as possible.

## Information on images used in the figures

**Table:** 

Figure	Database	Sequence	Image
Fig. 4A	LTIR	Saturated	00000043
Fig. 4B	LTIR	Saturated	00000044
Fig. 7A (row 1)	LTIR	quadrocopter2	00000858
Fig. 7A (row 2)	LTIR	hiding	00000023
Fig. 7A (row 3)	LTIR	crossing	00000058
Fig. 7A (row 4)	LTIR	street	00000056
Fig. 8A (row 1)	LITIV	SEQUENCE2	in000333
Fig. 8A (row 2)	LITIV	SEQUENCE5	in0003992
Fig. 8A (row 3)	LITIV	SEQUENCE8	in000105
Fig. 8A (row 4)	LITIV	SEQUENCE4	in000103
Fig. 9A (row 1)	OSU	00003	img_00008
Fig. 9A (row 2)	OSU	00006	img_00013
Fig. 9A (row 3)	OSU	00008	img_00013
Fig. 9A (row 4)	OSU	00005	img_00023
Fig. 10A (row 1)	Terravic	irw01	000272
Fig. 10A (row 2)	Terravic	irw10	000366
Fig. 10A (row 3)	Terravic	irw11	000970

## Supplemental Information

10.7717/peerj-cs.1064/supp-1 Supplemental Information 1 Source code and readme. The source code implements the Pedestrian Detection Algorithm.

## Additional Information and Declarations

### Data Availability

The following information was supplied regarding data availability: The data analyzed in this study are available at: - The Ohio State University (OSU) thermal pedestrian database (Davis & Keck, 2005): http://vcipl-okstate.org/pbvs/bench/Data/01/download.html, Collection 1 - 10 - The Polytechnique Montréal: LITIV dataset (Torabi, Massé & Bilodeau, 2012): https://www.polymtl.ca/litiv/en/codes-and-datasets, Thermal-visible registration (Torabi et al. dataset, CVIU, 2012 / St-Charles et al. CVPRW 2015), (Thermal) Sequence 1 - 9 - The Terravic Motion IR database (Miezianko, 2005): http://vcipl-okstate.org/pbvs/bench/Data/05/download.html, Outdoor Motion and Tracking Scenarios, Collection 1 - 10 - The Linkoping Thermal InfraRed (LTIR) dataset (Berg, Ahlberg & Felsberg, 2015): https://www.cvl.isy.liu.se/en/research/datasets/ltir/version1.0/ crossing, hiding, saturated, quadrocopter2, soccer, street
