# Supplementary figures and images for: A semi-automatic motion-constrained Graph Cut algorithm for Pedestrian Detection in thermal surveillance videos
Source: PeerJ Comput Sci. 2022 Sep 12;8:e1064. doi: 10.7717/peerj-cs.1064 (PMC9575872; doi:10.7717/peerj-cs.1064)

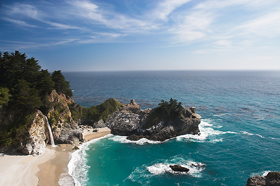

Supplement: Supplemental Information 1 — The source code implements the Pedestrian Detection Algorithm. [file peerj-cs-08-1064-s001.zip › cs-70932-GraphCutPeerJv2 JT staff/GraphCut_PeerJ_v2/maxflow/waterfall.bmp]

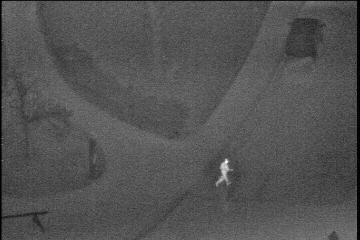

Supplement: Supplemental Information 1 — The source code implements the Pedestrian Detection Algorithm. [file peerj-cs-08-1064-s001.zip › cs-70932-GraphCutPeerJv2 JT staff/GraphCut_PeerJ_v2/MCGCE/img_00010.bmp]

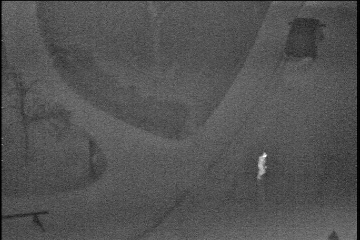

Supplement: Supplemental Information 1 — The source code implements the Pedestrian Detection Algorithm. [file peerj-cs-08-1064-s001.zip › cs-70932-GraphCutPeerJv2 JT staff/GraphCut_PeerJ_v2/MCGCE/img_00011.bmp]
